# Antibody desolvation with sodium chloride and acetonitrile generates bioactive protein nanoparticles

**DOI:** 10.1371/journal.pone.0300416

**Published:** 2024-03-14

**Authors:** Levi Collin Nelemans, Vinicio Alejandro Melo, Matej Buzgo, Edwin Bremer, Aiva Simaite

**Affiliations:** 1 R&D Center, InoCure s.r.o, Celákovice, Central Bohemian, Czech Republic; 2 Department of Hematology, University Medical Center Groningen/University of Groningen, Groningen, Groningen, The Netherlands; Cairo University, EGYPT

## Abstract

About 30% of the FDA approved drugs in 2021 were protein-based therapeutics. However, therapeutic proteins can be unstable and rapidly eliminated from the blood, compared to conventional drugs. Furthermore, on-target but off-tumor protein binding can lead to off-tumor toxicity, lowering the maximum tolerated dose. Thus, for effective treatment therapeutic proteins often require continuous or frequent administration. To improve protein stability, delivery and release, proteins can be encapsulated inside drug delivery systems. These drug delivery systems protect the protein from degradation during (targeted) transport, prevent premature release and allow for long-term, sustained release. However, thus far achieving high protein loading in drug delivery systems remains challenging. Here, the use of protein desolvation with acetonitrile as an intermediate step to concentrate monoclonal antibodies for use in drug delivery systems is reported. Specifically, trastuzumab, daratumumab and atezolizumab were desolvated with high yield (∼90%) into protein nanoparticles below 100 nm with a low polydispersity index (<0.2). Their size could be controlled by the addition of low concentrations of sodium chloride between 0.5 and 2 mM. Protein particles could be redissolved in aqueous solutions and redissolved antibodies retained their binding activity as evaluated in cell binding assays and exemplified for trastuzumab in an ELISA.

## Introduction

Around 30% of the FDA approved drugs in 2021 were therapeutic proteins and their approval for clinical use has, since 2014 till 2021, accounted for an average of 27% [1]. Currently, most commercial protein therapeutics are administered intravenously or subcutaneously and, due to low protein half-lives *in vivo*, often require frequent injections. Rapid protein clearance from the blood is caused by multiple factors such as physicochemical instability and enzymatic degradation [2,3]. Furthermore, most therapeutic protein targets are ubiquitously expressed, which can lead to off-target cell toxicity [4,5]. Thus, drug delivery systems (DDS) for proteins that preserve protein activity, provide sustained release, and passive or active targeting and, thus, toxicity reduction, are in demand [6–9]. Despite tremendous progress, DDS for therapeutic monoclonal antibodies (mAbs) are not yet clinically available. One of the challenges is that mAbs are administered in large doses (usually in doses above 300 mg), and any additional excipients needed for a DDS considerably increase the volume of the injection. This leads to an even greater burden to the patient when injected intravenously and the large volume often prevents subcutaneous injection all together [10]. Notably, most if not all DDS reported for the delivery of mAbs have a loading capacity (LC, percentage of the mass of the drug vs the mass of the DDS) below 7% [11–16].

One of the most common administration routes of mAbs is intravenous (IV) injection. However, often expression of the targeted antigen is not restricted to the therapeutic site, which can cause severe on-target, but off-site toxicity that limits their use [17,18]. As an alternative, several injectable polymer-based DDS have been suggested for local administration of mAbs [19,20]. However, such gel-like depots require large (> g20) needles that cause significant pain to patients [20,21] and require highly concentrated antibody/enzyme formulations to reach practical injection volumes (15 mL) [22,23]. As an example, rituximab for subcutaneous injection is 12 times more concentrated than the IV formulation [24]. Thus, IV injection is still the most prevalent method.

Various other types of IV protein DDS have been reported, such as polymeric nanoparticles (NPs) prepared by emulsion, liposomes, and exosomes [25]. Currently, the most common encapsulating method of mAbs is the water/oil/water double emulsion method [11,12,15,16]. The downside of this, and most other encapsulation methods, is the use of chemicals or techniques that can lead to significant protein denaturation [26–28]. Furthermore, this method often leads to low mAb loading. For instance, even after careful optimization by Varshochian et al. only a maximum LC of 7.1% could be achieved for bevacizumab [15]. Such LCs are impractical for therapeutic antibodies that tend to be administered in doses over 300 mg per injection [10]. Stepwise improvements in the process are unlikely to lead to 10-fold higher LCs. Therefore, novel strategies for the preparation of DDS need to be explored that either reduce the amount of excipients or increase the protein concentration for encapsulation.

Protein desolvation could be a promising technique to concentrate mAbs. Originally, protein desolvation was used as an alternative method to produce protein NPs without high stirring speeds or shearing forces (e.g. turax, sonication) and has been extensively researched as a non-toxic, biodegradable DDS [29–32]. In short, a water-miscible, non-solvent for proteins (e.g. ethanol) is drop-wise added into an aqueous protein solution under mild stirring. This leads to supersaturation of the proteins, resulting in the formation of protein NPs [31]. Various parameters influence the desolvation process, such as the protein characteristics (size, isoelectric point, net charge, etc.), initial protein concentration, temperature, and pH.

This process has mostly been studied on either human serum albumin [33–36] or bovine serum albumin [37–40]. However, the egg-white protein lysozyme and various plant-based proteins (such as zein, alginate, pea and soy proteins) are being investigated as alternative proteins for desolvation [41–43]. Normally, the protein particles serve as a DDS for the encapsulation of small molecules. To stabilize the protein particles and prevent redisolvation in aqueous solutions, they are either heat-treated or cross-linked. However, this process also alters the protein structure and reduces bioactivity. Protein desolvation has rarely been used as an intermediate step in the formation of active protein particles that themselves can be encapsulated in DDS. Therefore, very little is known about the activity after redissolving these protein particles. According to available literature, only Giteau et al. and Marales-Cruz et al. have shown remaining activity of peroxidase, beta-galactosidase, alpha-chemotrysin and lysozyme [44,45], whereas Nelemans et al. has shown remaining enzymatic activity of amylase after desolvation [46]. If mAb desolvation is also non-detrimental to mAb activity, this process can be used as a stable intermediate to concentrate and encapsulate mAbs inside DDS.

In this paper, the feasibility of using protein desolvation to prepare protein NPs of commercial therapeutic antibodies is investigated. Specifically, controllable protein particles sizes with high yield (∼90%), high remaining binding activity after protein desolvation and redissolution (*>*80%) and the influence of low concentrations (<2 mM) of sodium chloride (NaCl) on protein particle size, yield and remaining activity are shown. Such concentrated therapeutic protein NPs could then potentially be used for the development of protein-based DDS of high LC.

## Materials and methods

### Materials

All monoclonal antibodies (atezolizumab (tecentriq), cetuximab (erbitux), daratumumab (darzalex), elotuzumab (empliciti), rituximab (truxima), trastuzumab (herzuma)) were kindly provided by the pharmacy of the University Medical Center in Groningen (UMCG, Groningen, the Netherlands). Goat anti-human IgG biotin (PAB10694) was obtained from Abnova (Taipei, Taiwan). Human HER2 with his-tag (10126-ER) and recombinant human Fc gamma RIIIA alexa fluor 647 protein (AFR4325-020) were purchased from R&D systems (Minneapolis, United States). Tween-20 (polysorbate-20) (P9416) from Sigma (Saint Louis, United States). 1-step Turbo TMB-ELISA (34022), High sensitivity streptavidin-HRP (21130) and nickel-coated 96 wells plates (15442) were obtained from ThermoFisher (Waltham, United States). Finally, 100 kDa Spectra-Por Float-A-Lyzer G2 (734–3576), PBS 10x (K813), Acetonitrile (HiPerSolv, 83640), NaCl (GRP RECTAPUR, 27800), water (GRP RECTAPUR, 83612), and Sodium dodecyl sulphate (442442F) were purchased from VWR (Radnor, United States). OVCAR-3, ES-2, U2932, MDA-MB-231 and SK-BR-3 were purchased from the American Type Culture Collection (ATCC, Manassas, United States).

### Dialysis of monoclonal antibodies

The commercial monoclonal antibodies were dialyzed against water for 36 hours at 4°C containing 1 mg/mL polysorbate-20 using a 100 kDa Spectra-Por Float-A-Lyzer G2 (Spectrum laboratories/Repligen, Waltham, United States). After dialysis, the concentration was measured using BCA assay (ThermoFisher, Waltham, United States) according to manufacturer’s recommendations. Absorbance was measured at 562 nm (microplate reader, SpectraMax, Molecular Devices, San Jose, United States).

### Protein nanoprecipitation

The monoclonal antibody precipitation protocol was adapted from [45,46]. In short, 200 μL of 10 mg/mL monoclonal antibody with various amounts of NaCl (0–2 mM), was precipitated by dropwise addition (0.5 mL/min, G18 needle) of 1.1 mL of acetonitrile (5.5:1 ACN:water) under stirring (1300 rpm). After addition, the samples were incubated for 5 min at 20°C, before their size and distribution was characterized using DLS (NanoPhox, Sympatec, Clausthal-Zellerfeld, Germany).

### Protein precipitation yield

1 mL of the obtained protein NPs were spun down at 22,000 RCF for 90 min. 100 μL of the supernatant was diluted with 900 μL of water and the protein concentration was determined using standard microBCA assay (ThermoFisher, Waltham, United States) protocol. Absorbance was measured at 562 nm (microplate reader, SpectraMax, Molecular Devices, San Jose, United States) and the yield was calculated as follows:

$$\text{Yield}\;\text{(\%)}=\frac{\text{theoretical}\;\text{maximum}\;\text{concentration}\;-\text{measured}\;\text{concentration}}{\text{theoretical}\;\text{maximum}\;\text{concentration}}\times 100$$

The theoretical maximum concentration is calculated by correcting the starting protein concentration (10 mg/mL) with the relevant dilutions of the assay, and thus represents the maximum concentration possible if all protein stayed in solution.

### Protein redisolvation yield

1 mL of the obtained protein NPs were added to 14 mL of PBS containing 0.5 mg/mL polysorbate-20 and the solution was spun down (22,000 RCF) for 90 min. The protein concentration was determined using standard microBCA assay (ThermoFisher, Waltham, United States) protocol. Absorbance was measured at 562 nm (microplate reader, SpectraMax, Molecular Devices, San Jose, United States) and the redisolvation yield was calculated as follows:

$$\text{Redissolved}\;\text{(\%)}=\frac{\text{measured}\;\text{concentration}}{\text{theoretical}\;\text{maximum}\;\text{concentration}}\times 100$$

The theoretical maximum concentration is calculated by correcting the starting protein concentration (10 mg/mL) with the relevant dilutions of the assay, and thus represents the maximum concentration possible if all protein stayed in solution. To further confirm redisolvation, 1 mL of the obtained protein NPs were added to either 14 mL ACN/water (5.5:1 ACN:water) or PBS containing 0.5 mg/mL polysorbate-20, and measured on DLS.

### Binding activity of redissolved monoclonal antibodies

The binding activity of mAbs after desolvation was analyzed using an ELISA and cell binding assay. For the ELISA, the 96-wells, Ni-coated plate was washed with washing buffer (3x)(0.05% polysorbate-20 in PBS), between each step. All samples, except HER2, were diluted in blocking buffer (1% BSA in washing buffer) before use. Finally, all samples were incubated for 1 hour at 37°C between each step, unless stated otherwise. In short, (1) 100 μl of 100 ng/mL recombinant HER2 was added to a 96 wells Ni-plate and incubated overnight at 4°C. (2) The wells were blocked with 200 μl blocking buffer. (3) 100 μl Trastuzumab standards (656.25–21,000 pg/mL), samples and controls were added. (4) 100 μl of 1000 ng/mL monoclonal goat anti-human-biotin was added. (5) 100 μl of 10,000x diluted HRP-strep was added and samples were washed 5x after incubation. (6) 100 μl TMB medium was added and the reaction was stopped after 15 min with 100 μL 2 M sulfuric acid. Finally, the absorbance was measured at 450 nm using a microplate reader (SpectraMax, Molecular Devices, San Jose, United States). The activity was calculated as follows:

$$\text{Activity}\;\text{(\%)}=\frac{\text{measured}\;\text{concentration}\;\text{ELISA}}{\text{theoretical}\;\text{maximum}\;\text{concentration}}\times 100$$

The theoretical maximum concentration is calculated by correcting the starting protein concentration (10 mg/mL) with the relevant dilutions of the assay, and thus represents the maximum concentration possible if all protein stayed in solution and has binding activity.

For the cell binding studies, 0, 0.01, 0.1, 1, 10 and 15 μg/mL of trastuzumab, atezolizumab or daratumumab solutions were added to 1x10^4^ OVCAR-3, ES-2, U2932, MDA-MB-321.(HER2) or SK-BR-3 cells, respectively. Samples were incubated for 30 min at 4°C and afterwards washed with 5 mL of PBS. Then, 1 μL of Mouse anti-human IgG-PE was added and incubated for 30 min at 4°C. Alternatively, cells were incubated with 2 μg/mL of Alexa 647 labelled, recombinant Fc Receptor for 30 min at 4°C. Cells were washed (2x) with 5 mL of PBS and the MFI of PE or Alexa 647 was measured using a flow cytometer (Cytoflex, Beckman coulter, Brea, United States). The MFI was normalized, with the highest intensity corresponding to 100% and 0 μg/mL as 0% binding, respectively.

### Dynamic light scattering

Protein particles were analyzed by dynamic light scattering (DLS) (NanoPhox, Sympatec, Clausthal-Zellerfeld, Germany) and measured directly as suspension in their ACN:water mixture with the following settings: 25°C, refraction 1.35, viscosity 0.53 mPas. The viscosity settings were verified by measuring known 100 nm polystyrene particles (43302, Sigma) in the same ACN:water mixture. The z-average was used to report the average particle size. Samples were diluted if the observed kilocounts per second (KCPS) exceeded 500. Further data analysis was performed using Python 3.0.

### Statistical analysis

Data consists of at least three independent experiments and is presented as mean ± SD with a *p<0.05, **p<0.005, ***p<0.0005, ****p<0.0001 considered as statistically significant. Statistical analysis was performed with GraphPad Prism V9.1.0 (GraphPad Software Inc., USA). Normality of distribution was checked with the Shapiro-Wilk normality test. To determine statistical significance of the desolvation yield, redissolution and activity, a paired, Geisser-Greenhouse corrected, one-way ANOVA was performed and, if significant (p<0.05), was followed by a Tukey Kramer post-hoc test. Differences between trastuzumab, atezolizumab and daratumumab binding curves were analyzed as follows: Using GraphPad Prism, a non-linear fit (specific binding with Hill slope) of each individual binding curve was generated and the Bmax, h and Kd values were subjected to a T-test, as recommended by [48].

## Results

Six commercially available mAbs were precipitated; atezolizumab (ATZ), cetuximab (CTX), daratumumab (DARA), elotuzumab (ELO), rituximab (RTX), and trastuzumab (TRA), and the influence of the process parameters on size, protein particle yield, and activity of the proteins was investigated. The known physicochemical properties of the antibodies, as well as the composition of their commercial formulations, are summarized in Table 1.

**Table 1:** Physicochemical properties and excipients of the commercial monoclonal antibodies atezolizumab, cetuximab, daratumumab, elotuzumab, rituximab and trastuzumab.

Antibody	Half-life (days)	Mw (kDa)	Isoelectric point [47]	Concentration (mg/mL)	Target	Type	Excipients
**Atezolizumab**	27	145	8.6	60	PDL1	humanized	polysorbate 20, sucrose, glacial acetic acid, L-histidine
**Cetuximab**	4.75	146	8.8	5	HER1	chimeric	polysorbate 80, sodium chloride, citric acid monohydrate, sodium hydroxide, glycine
**Daratumumab**	18	145	-	20	CD38	humanized	polysorbate 20, mannitol, sodium chloride, sodium acetate trihydrate, glacial acetic acid
**Elotuzumab**	-	148	8	25	SLAMF7	humanized	polysorbate 80, sucrose, citric acid monohydrate, sodium citrate
**Rituximab**	22	144	9.4	10	CD20	chimeric	polysorbate 80, sodium chloride, sodium citrate
**Trastuzumab**	28	146	9.1	21	HER2	humanized	polysorbate 20, a,a-threhalose dihydrate, L-histidine hydrocloride monohydrate, L-histidine

### Antibody desolvation process optimization

**Fig 1:**
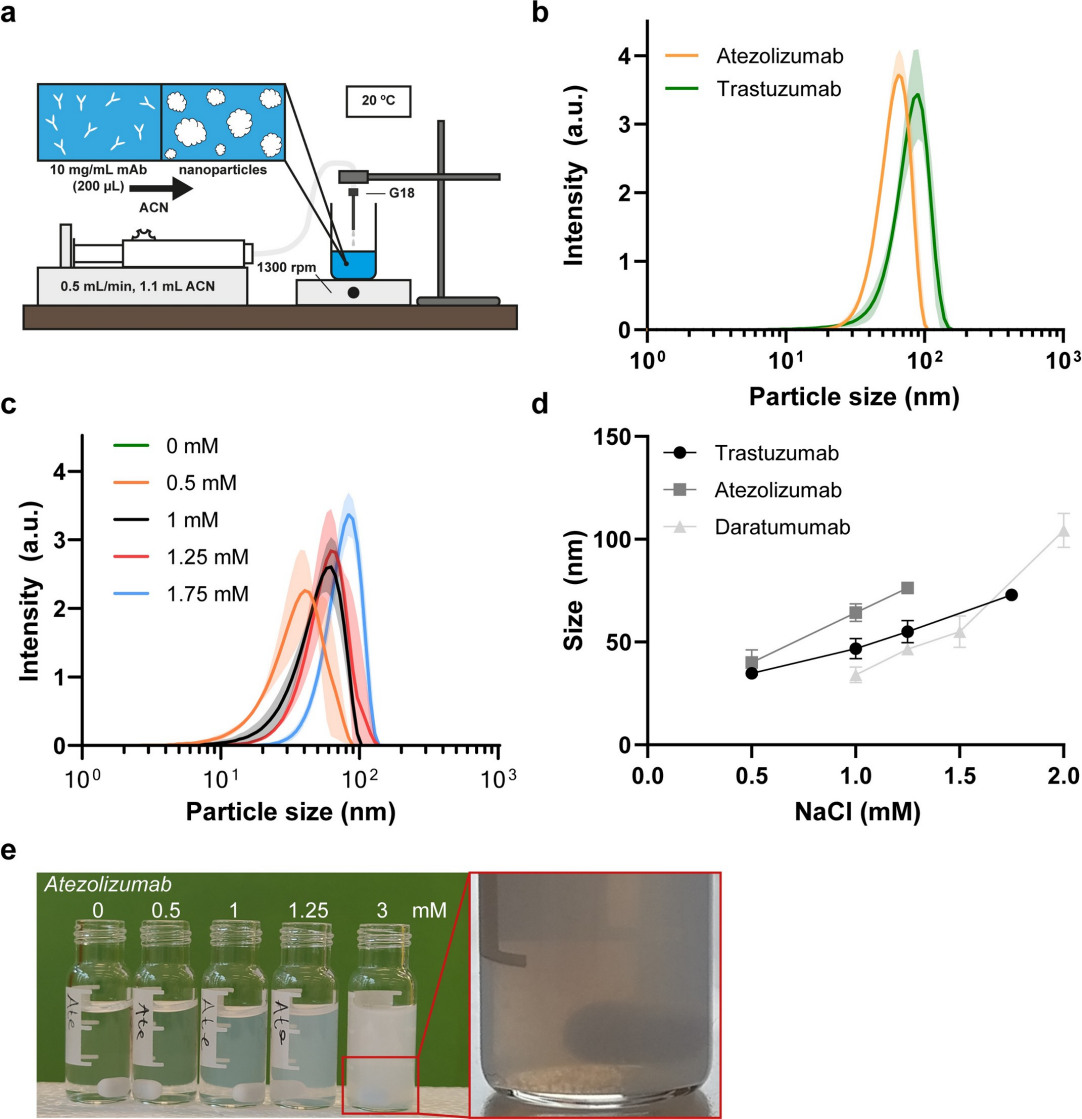
Effect of sodium chloride on monoclonal antibody desolvation particle size. (a) Schematic illustration of the monoclonal antibody (mAb) desolvation process in which 1.1 mL of acetonitrile was drop-wise added (0.5 mL/min) to 200 μL of mAb (10 mg/mL) at 20°C under stirring (1300 rpm). (b) Dynamic light scattering (DLS) intensity distribution of desolvated commercial mAbs nanoparticles atezolizumab and trastuzumab (n = 4). The mAbs elotuzumab, rituximab, daratumumab and cetuximab flocculated out of solution. (c) DLS intensity distribution of desolvated trastuzumab at various sodium chloride (NaCl) concentrations (n = 3). (d) Influence of initial NaCl concentration on particle size after desolvation of trastuzumab, atezolizumab and daratumumab, with corresponding Table 2 (n = 3). The solid lines are guides to the eye. (e) Desolvated atezolizumab at indicated NaCl concentrations, including flocculated particles at 3 mM. Data are presented as mean with all shaded areas and error bars denoting standard deviation.

The six commercial antibodies were desolvated using a protocol first reported by Morales-Cruz et al. [45] and further optimized by Nelemans et al. [46]. Briefly, the anti-solvent acetonitrile (ACN) was drop-wise added to 10 mg/mL antibody solution at a 0.5 mL/min rate under stirring (1300 rpm) to reach a 5.5:1 ACN:water ratio (Fig 1A). All experiments were performed in a temperature stabilized room (20°C), as temperature has a major influence on process reproducibility.

Only the commercial antibodies TRA and ATZ could be precipitated into protein NPs with an average particle size of 88 ± 3 nm and 66 ± 2 nm, respectively, as determined by dynamic light scattering (DLS) (Fig 1B). Furthermore, the TRA particles remained stable in solution after 30 and 60 minutes of storage, although a slight size increase was observed (A in S1 Fig). The other four mAbs (ELO, RTX, DARA, and CTX) flocculated out of solution upon addition of ACN. Lowering the concentration of the other mAbs to 5 and 1 mg/mL did not result in stable protein NPs. It was previously suggested by Tarhini et al. [39] that the desolvation process is influenced by the net-charge of the protein. Since the mAbs all had similar physicochemical properties (Table 1), the excipients in the commercial mAb formulation might interfere with the surface properties of these antibodies and, hereby, influence desolvation. To investigate this possibility, TRA, DARA and ATZ were dialyzed against deionized water with 1 mg/mL of the surfactant polysorbate-20 to remove the potentially interfering excipients. Interestingly, when the mAbs TRA, DARA, and ATZ were desolvated after dialysis, no turbidity was observed nor particle size detected on DLS, which might suggest that one or more of the excipients induced desolvation.

Indeed, all four mAbs that initially could not be precipitated (CTX, RTX, DARA, ELO), contained the salts NaCl (3 out of 4) and/or sodium citrate (3 out of 4), whereas TRA and ATZ did not (Table 1). As protein desolvation is expected to be influenced by the surface charge of the protein, addition of salt in the solution may shield this charge and thus affect the process.

### Protein nanoparticle size is controlled by addition of salt

To test the influence of salt on protein desolvation, TRA, DARA and ATZ were dialyzed and desolvated with varying starting concentrations of NaCl (0.5–2.0 mM) in their solution. First, desolvation of TRA containing 0.5 mM NaCl led to a protein particle size of 35 ± 2 nm, whereas without addition of salt, no protein particles were detected with DLS (Fig 1C and Table 2). A further increase in salt concentration to 1 mM, 1.25 mM and 1.75 mM increased the protein particle size to 47 ± 5 nm, 55 ± 5 nm and 73 ± 2 nm, respectively (Fig 1C and Table 2). Furthermore, the polydispersity index (PDI) of the trastuzumab particles stayed below 0.2 for all salt concentrations (0.06 ± 0.09, 0.17 ± 0.02, 0.13 ± 0.02, 0.12 ± 0.01 for 0.5, 1, 1.25, 1.75mM NaCl, respectively. Table 2).

**Table 2:** Influence of initial sodium chloride concentration on particle size and polydispersity index after desolvation of trastuzumab, atezolizumab and daratumumab.

	Atezolizumab		Daratumumab		Trastuzumab	
NaCl (mM)	Z-average (nm)	PDI	Z-average (nm)	PDI	Z-average (nm)	PDI
**0.5**	40±6	0.18±0.11			35±2	0.06±0.09
**1**	64±4	0.12±0.02	34±4	0.10±0.08	47±5	0.17±0.02
**1.25**	76±2	0.13±0.01	47±1	0.16±0.02	55±5	0.13±0.02
**1.5**			55±8	0.16±0.03		
**1.75**					73±2	0.12±0.01
**2**			104±8	0.20±0.01		

Data are presented as mean with standard deviation (n = 3). Abbreviations: NaCl ‐ sodium chloride, PDI ‐ polydispersity index.

For both DARA and ATZ, an increase in particle size with increasing NaCl concentration was detected and regardless of the amount of NaCl, the PDI of ATZ and DARA remained below 0.2 (Table 2). Interestingly, the rate of size increase as well as the minimum amount of NaCl required to measure nanoparticles on DLS differed for the three antibodies (Fig 1D). For example, no protein particles were measured by DLS until 0.5 mM NaCl was added to TRA and ATZ (35 ± 2 nm and 40 ± 6 nm, respectively), whereas DARA required 1 mM NaCl (34 ± 4 nm). Furthermore, an increase in particle size increased the observed turbidity (Fig 1E). Finally, for all three mAbs, initial salt concentrations past 2 mM led to larger, unstable particles that flocculated out of solution during or shortly after ACN addition with a more polydisperse character and visible, larger aggregates (Fig 1E, atezolizumab 3 mM).

In conclusion, the size of the protein particles could be controlled by adjusting the amount of NaCl in the initial mAb solution. However, there appears to be a limit to the lowest and highest amount of NaCl that can be added that will lead to stable and observable mAb particles. Further, protein desolvation using ACN may negatively affect the stability of the therapeutic proteins. Therefore, precipitation yield, redissolution capability, and activity of precipitated proteins were investigated and are discussed next.

### Desolvation yield

**Fig 2:**
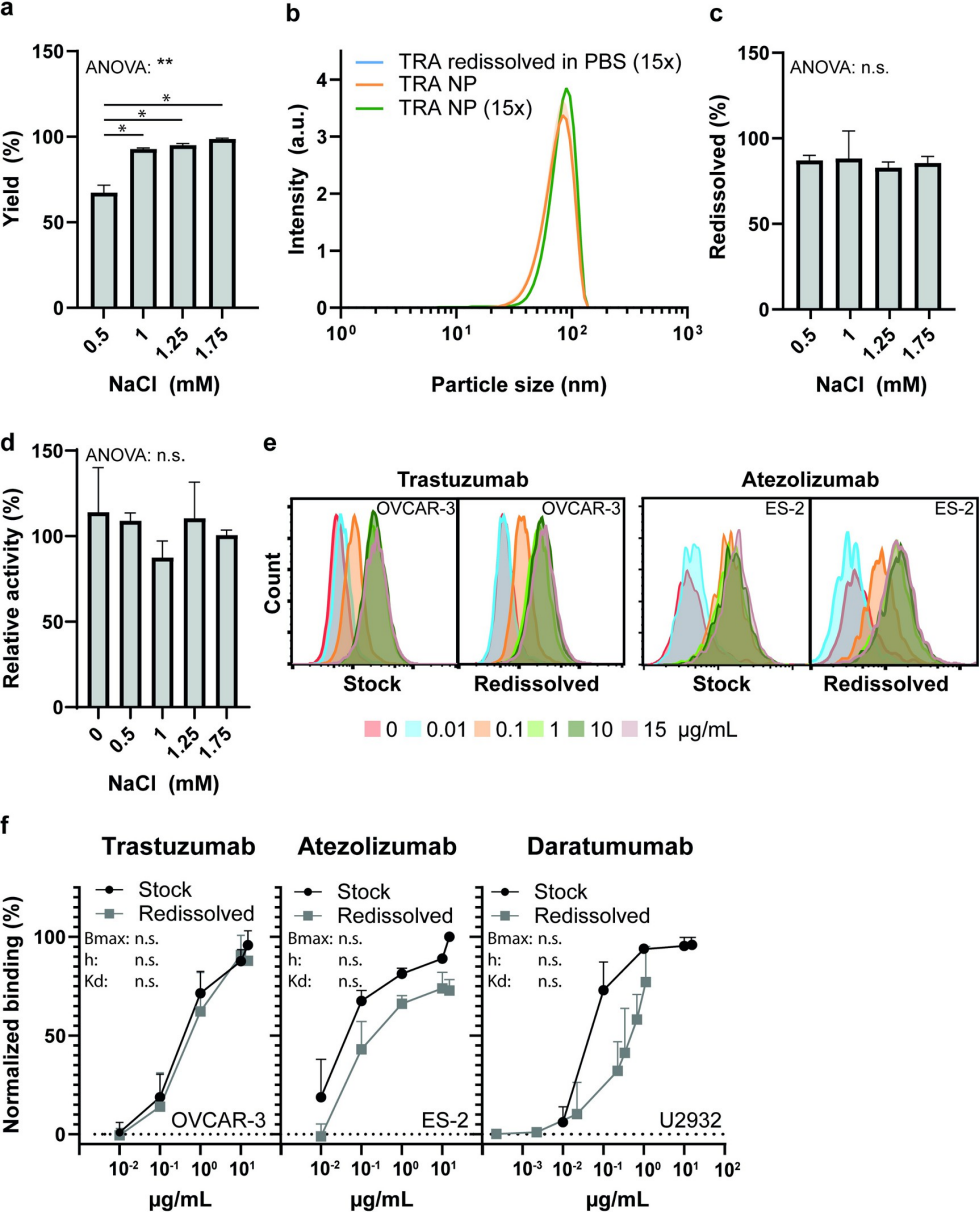
Yield, redissolution and activity of desolvated monoclonal antibodies. (a) Yield of trastuzumab desolvation with different sodium chloride concentrations. (n = 3) (b) DLS intensity distribution of redissolved and diluted trastuzumab nanoparticles (n = 3). (c) Percentage of trastuzumab nanoparticles that redissolved in PBS with 0.5 mg/mL polysorbate-20 (n = 3). (d) Relative activity of redissolved trastuzumab compared to dialyzed trastuzumab (prior to desolvation) in a HER2 specific ELISA (n = 3). (e) Representative histograms of redissolved and stock trastuzumab and atezolizumab binding to OVCAR-3 and ES-2 cells, respectively. (f) Binding study of redissolved and stock trastuzumab, atezolizumab and daratumumab to OVCAR-3, ES-2 and U2932 cells, respectively. The solid lines are guides to the eye (n = 3). Data are presented as mean with all shaded areas and error bars denoting standard deviation. Statistical analyses were performed using a paired, one-way Anova, followed by a Tukey’s test if significant (p<0.05). Differences between trastuzumab, atezolizumab and daratumumab binding curves were analyzed by subjecting the Bmax, h and Kd values of each binding curve to a T-test as recommended by [48]. “*” and “**” indicates p<0.05 and p<0.005, respectively.

An important factor when preparing typically expensive protein therapeutics is the yield. The desolvation yield of TRA particles was NaCl concentration-dependent and ranged from 67 ± 4% with 0.5 mM NaCl to 98.7 ± 0.6% with 1.75 mM NaCl (Fig 2A). Notably, the desolvation yield was significantly lower at 0.5 mM NaCl, compared to 1, 1.25 and 1.75 mM NaCl (92.9 ± 0.6%, 95 ± 1%, and 98.7 ± 0.6%, respectively). However, when the supernatant was analyzed by DLS after centrifugation, some particles could still be observed, thus potentially not all particles were spun down. Therefore, the calculated yield at 0.5 mM NaCl might be an underestimation. In conclusion, the yield of the protein particles is high (*>* 90%) regardless of NaCl concentration (1–1.75 mM).

### Protein activity after desolvation

Finally, the binding activity of various mAb particles after being redissolved into aqueous solution were investigated. Hereto, the mAb NPs were added to PBS with 0.5 mg/mL polysorbate-20 or ACN:water solution (5.5:1) to yield a 15-fold dilution. No trastuzumab NPs could be observed after addition to PBS, whereas mAb NPs remained intact after dilution in the ACN:water solution (1.75 mM NaCl, Fig 2B). Furthermore, after centrifugation of the PBS solution (to spin down potential undissolved particles), almost all protein was still observed in the supernatant (87%, 88%, 82%, 85% for 0.5, 1,1.25 and 1.75 mM NaCl, respectively), indicating a near complete redissolution (Fig 2C).

An ELISA-based assay detected similar theoretical binding activities of redissolved TRA to HER2 (80–100%) for all NaCl concentrations (Fig 2D). Additionally, redissolved TRA selectively bound to MDA-MB-231 cells expressing HER2 and could be detected by a recombinant Fc receptor (S1B and S1C Fig). Furthermore, both redissolved TRA, ATZ and DARA (previously desolvated with 1 mM NaCl) bound respectively to the cell lines OVCAR-3, ES-2 and U2932 in a concentration-dependent manner, not significantly different from their commercial stock mAbs (Fig 2E and 2F), indicating retention of bioactivity.

In conclusion, three different commercial antibodies were desolvated into protein NPs with high yield. Their size could be precisely controlled by the addition of low amounts of NaCl (0.5–2 mM). This protein desolvation process appears to be non-detrimental to the protein structure of TRA, ATZ and DARA that all retained antigen-specific binding activity after being redissolved in PBS with 0.5 mg/mL polysorbate-20.

## Discussion

### Mechanism behind protein desolvation

The results presented in this paper demonstrate that therapeutic antibodies can be precipitated into nanoparticles using the desolvation method, by first dialyzing the antibodies in water with 1 mg/mL polysorbate-20. Furthermore, their particle size can be controlled by the addition of low concentrations of NaCl (<2 mM), while retaining binding activity after being redissolved.

Protein desolvation is a complex process, but the mechanisms behind protein stabilization, precipitation and denaturation upon addition of agents (salts, solvents, etc.) were already described by Timasheff et al. [49] in 1993. In case of protein desolvation by organic solvents, the driving force behind desolvation is the reduction of the hydration layer of water molecules around the proteins. This layer has a dampening effect on the attractive forces between the proteins, and thereby prevents precipitation in aqueous solutions. However, with increasing concentrations of non-solvent, the water at the protein surface will be displaced by the non-solvent. A reduction of the hydration layer leads to increased attractive forces between the proteins, which can induce precipitation.

Some of these attractive and repulsive forces between proteins or particles can be described by the Derjanguin-LandauVerwey-Overbeek (DLVO) theory [50]. The DVLO theory in Eq 1, describes the total potential interaction energy (W_total_(D)) between two particles in a liquid, consisting of both the Van der Waals W_a_(D) (attractive) and electrostatic (electric double layer, repulsive) W_r_(D) forces.


$$W_{total}(D)=W_{a}(D)+W_{r}(D)$$ (1)

**Fig 3:**
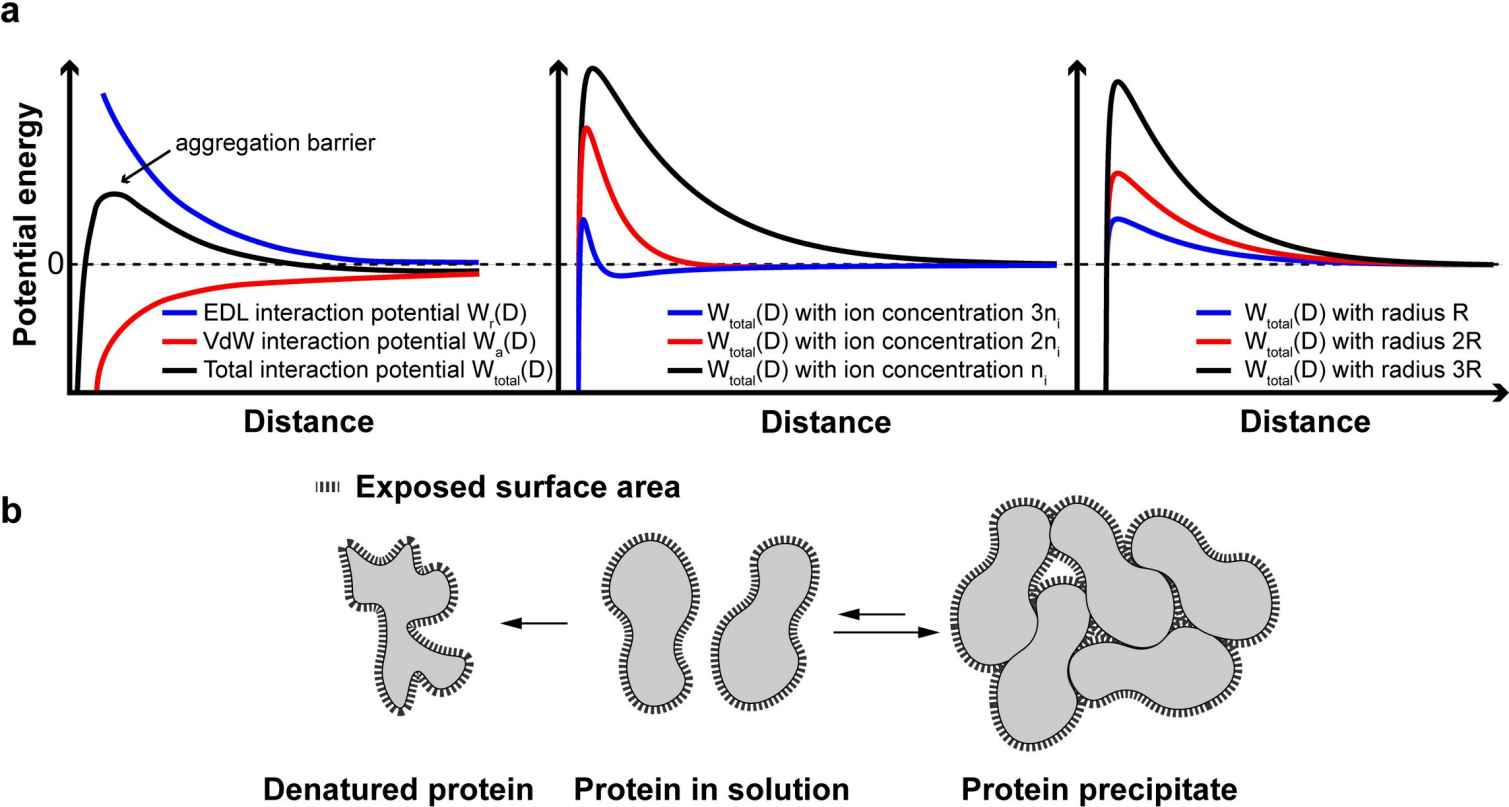
DLVO and desolvation theory. (a) Schematic depiction of the interplay between the potential energy of electrostatic (EDL) and Van der Waals (VdW) forces over distance and the influence of ion concentration (n) and particle radius (R) on the aggregation barrier. (b) Schematic illustration of exposed protein surface area to the solvent when denatured or precipitated. In general, protein precipitation leads to reduced protein surface area, whereas denatured protein increases its surface area.

This equation can be rewritten to describe the potential energy between two identical particles with radius R in close proximity and is described in more detail by Polte et al. [51] (Eq 2):

$$W_{total}(D)=-\frac{AR}{12D}+2\pi \varepsilon \varepsilon_{0}R\Psi_{\delta }^{2}exp(-\kappa D)$$ (2)

with κ as the Debye constant:

$$\kappa ={\left[\sum_{i}\frac{z_{i}^{2}e^{2}n_{i\infty }^{2}}{k_{B}T}\right]}^{2}$$ (3)

with Hamaker constant A, distance between the two particles D, permittivity of vacuum ε, dielectric constant ε_0_, electric surface potential Ψ, elemental charge e, valency z and concentration of ions n, Boltzmann constant k_B_, and temperature T. Plotting the potential energy versus the particle distance will show a maximum energy (the aggregation barrier) which needs to be overcome for two particles to coagulate (Fig 3A, left). If this energy cannot be overcome, the particles will remain stable in solution. Polte et al. [51] shows that especially the electrostatic term is affected by different assumptions, such as the ionic concentration, particle size and surface potential.

In the case of ACN desolvation with low concentrations of NaCl, the aggregation barrier is lowered in two ways, 1) by increasing ionic concentration (increase in n decreases W_r_(D), Fig 3A, middle) 2) and by lowering the dielectric constant ε_0_ with increasing ACN concentration in ACN-water mixtures [52]. Counteracting this reduction in the aggregation barrier is the particle size R, in which coagulation of two larger particles requires more energy than smaller particles (higher aggregation barrier) (Fig 3A, right).

After initial particle formation, the protein particles were hypothesized to coagulate and increase in size, until the required energy to overcome the aggregation barrier would exceed the available energy in the system (controlled by temperature, stirring speed, etc.). This theory could explain why at lower sodium chloride concentrations (< 0.5 mM) no particles were observed (aggregation barrier > system’s energy) or why at higher sodium chloride concentrations the particles all flocculated out of solution (aggregation barrier < system’s energy). In other words, the salt lowered the aggregation barrier to such an extent that the particles could not be stabilized.

This theory is further supported by observations of von Strop et al. [34], which showed some control on protein particle size (170–300 nm) by changing the desolvating agent (dielectric constant). Other methods for controlling particle size have also previously been shown, e.g. by changing the pH (protein surface potential) or ionic strength [37,39]. However, all these methods were more limiting and yielded less precise size control than the addition of low concentrations of NaCl identified here.

Clearly, desolvation with a combination of salt and organic solvent has a synergistic effect, as very low concentrations of NaCl (0.5–2 mM) influenced desolvation, compared to salting out (1–5 M). In line with this, precipitation with 1 to 30 mM of NaCl combined with acetone (50–80%) was previously reported to maximize protein precipitation recovery [53–56]. It was proposed that ionic pairing between Na+, Cl- and charges on the protein might be responsible for this effect. Whereas ionic pairing is uncommon in water, this effect becomes more relevant in solvents with low bulk permittivity (such as ACN). Thus, ionic pairing would shield the protein’s charge, thereby, increasing hydrophobic interactions between the proteins or, in other words, lower the aggregation barrier [53]. This would subsequently lead to larger protein particles after desolvation.

### Protein activity after desolvation

Previous studies that precipitated mAbs with salts and polymers, a method often used as an alternative to protein A purification, showed that precipitation does not affect mAb activity. For example, Matheus et al. [57] used IgG precipitation (with salts and polymers, e.g. polyethylene glycol) as an up-concentration method and reported full activity after redissolving the precipitate. Retaining activity after protein precipitation with organic solvents is less investigated, however Tscheliessnig et al. used ice cold ethanol precipitation to purify IgGs with high yield and without aggregates after redissolution [58].

Initially, the antibodies at the interface with the organic solution were hypothesized to have a higher chance of being irreversibly denatured as commonly observed in water/oil/water emulsions [59–61]. Thus, the remaining activity after redissolution should increase with protein particle size as more proteins would be shielded in the core of the protein particles. Nevertheless, no discernible difference in mAb activity was observed between the different sizes of protein NPs, nor did activity significantly differ from the stock mAb. Indeed, previous reports confirm that enzymatic activity of lysozyme, alpha-chymotrypsin and amylase were unaffected by desolvation with ACN [45,46]. Furthermore, no structural changes were observed after redissolution of desolvated lysozyme, alpha-chymotrypsin, and beta-galactosidase by glycofurol [44]. According to Timasheff et al. [49] whether protein desolvation leads to protein denaturation, depends on the properties of the non-solvent. If the non-solvent (e.g. ACN) has a higher affinity for the protein than water, especially the hydrophobic groups within the protein, protein denaturation and unfolding can occur (Fig 3B, left). If the non-solvent prefers to be excluded from the protein surface, the protein will precipitate out of solution, thereby limiting the surface area exposed to the non-solvent (Fig 3B, right). This conformation is more energetically favorable, hence the spontaneous nature of precipitation. Sirotkin et al. investigated binding of ACN to lysozyme at various ratios [62] and showed that at most water concentrations (0.2–0.8 water/ACN), lysozyme is indeed enriched with water, strengthening this hypothesis. However, without investigation of the conformation of mAbs, partial but reversible denaturation during desolvation with ACN cannot be ruled out.

### Applications of mAb NPs

The aim of this research was to create stable, highly concentrated protein particles which could be used in various drug delivery systems. For example, the obtained mAbs nanoparticles could be coated with polymers such as poly(lactic-co-glycolic acid) (PLGA), as previously described by Morales-Cruz et al. [45] and Giteau et al. [44]. Thereby, mAb-loaded PLGA nanoparticles with a slow and sustained release profile can be generated. Future work will focus on the effects of mAb particle size on encapsulation efficiency, loading capacity, PLGA particle size and sustained release. Additionally, the stability and purity of redissolved mAbs will be further evaluated, as degraded proteins can enhance immunogenicity upon administration [63].

Furthermore, subcutaneous injections of high concentrations (> 150 mg/mL) of mAbs are limited by the viscosity of the solution. Currently, mAb suspensions are being investigated as a means to lower the viscosity [64]. For example, the monoclonal antibodies RTX and DARA as well as an anti-PSMA/CD3 bispecific antibody have already been successfully encapsulated in in-situ forming depots for sustained release [65,66]. However, the current limitation of this approach is that antibodies need to be spray dried, which can reduce bioactivity, requires optimization, large amounts of energy and time, whereas desolvation with solvents like ACN provide a more facile, cheap and rapid alternative. Notably, if nanoprecipitation could also be achieved with solvents such as N-Methyl-2-pyrrolidone (NMP), which is deemed suitable for injection, then high concentrations of protein could be precipitated, mixed with the correct polymers and directly injected to create in-situ forming gels [67].

## Conclusions

The commercial antibodies trastuzumab, daratumumab and atezolizumab could be desolvated into protein nanoparticles with high yield (*>*80%) after dialysis in water with 1 mg/mL polysorbate-20. Furthermore, the formed protein nanoparticles could be redissolved in aqueous solutions without affecting binding activity. Finally, low concentrations of NaCl (0.5 to 2 mM) were used to precisely control mAb particle size, without influencing mAb activity.

## Supporting information

S1 Fig Stability desolvated particles over time and cellular binding of redissolved trastuzumab. **(a)** Dynamic light scattering (DLS) intensity distribution of desolvated trastuzumab particles 0 min (n = 4), 30 min (n = 2) and 60 min (n = 2) after production. **(b)** Binding study of redissolved and stock trastuzumab to SK-BR-3 cells, detected by recombinant FcR (n = 1). **(c)** Histogram of redissolved and stock trastuzumab binding MDA-MB-231 and MDA-MB-231.HER2 cells (n = 1). Data are presented as mean with all shaded areas denoting standard deviation.
(PDF)

S1 File Numerical data used for plotting figures. (XLSX)
