# Supplementary material for: Antibody desolvation with sodium chloride and acetonitrile generates bioactive protein nanoparticles
Source: PLoS One. 2024 Mar 14;19(3):e0300416. doi: 10.1371/journal.pone.0300416 (PMC10939210; doi:10.1371/journal.pone.0300416)
Supplement: S1 Fig — (a) Dynamic light scattering (DLS) intensity distribution of desolvated trastuzumab particles 0 min (n = 4), 30 min (n = 2) and 60 min (n = 2) after production. (b) Binding study of redissolved and stock trastuzumab to SK-BR-3 cells, detected by recombinant FcR (n = 1). (c) Histogram of redissolved and stock trastuzumab binding MDA-MB-231 and MDA-MB-231.HER2 cells (n = 1). Data are presented as mean with all shaded areas denoting standard deviation. (PDF) [file pone.0300416.s001.pdf]

Supplementary Information

Antibody desolvation with sodium chloride and acetonitrile generates bioactive protein nanoparticles

Levi Collin Nelemans, Vinicio Alejandro Melo, Matej Buzgo, Edwin Bremer, and Aiva Simaite

S1

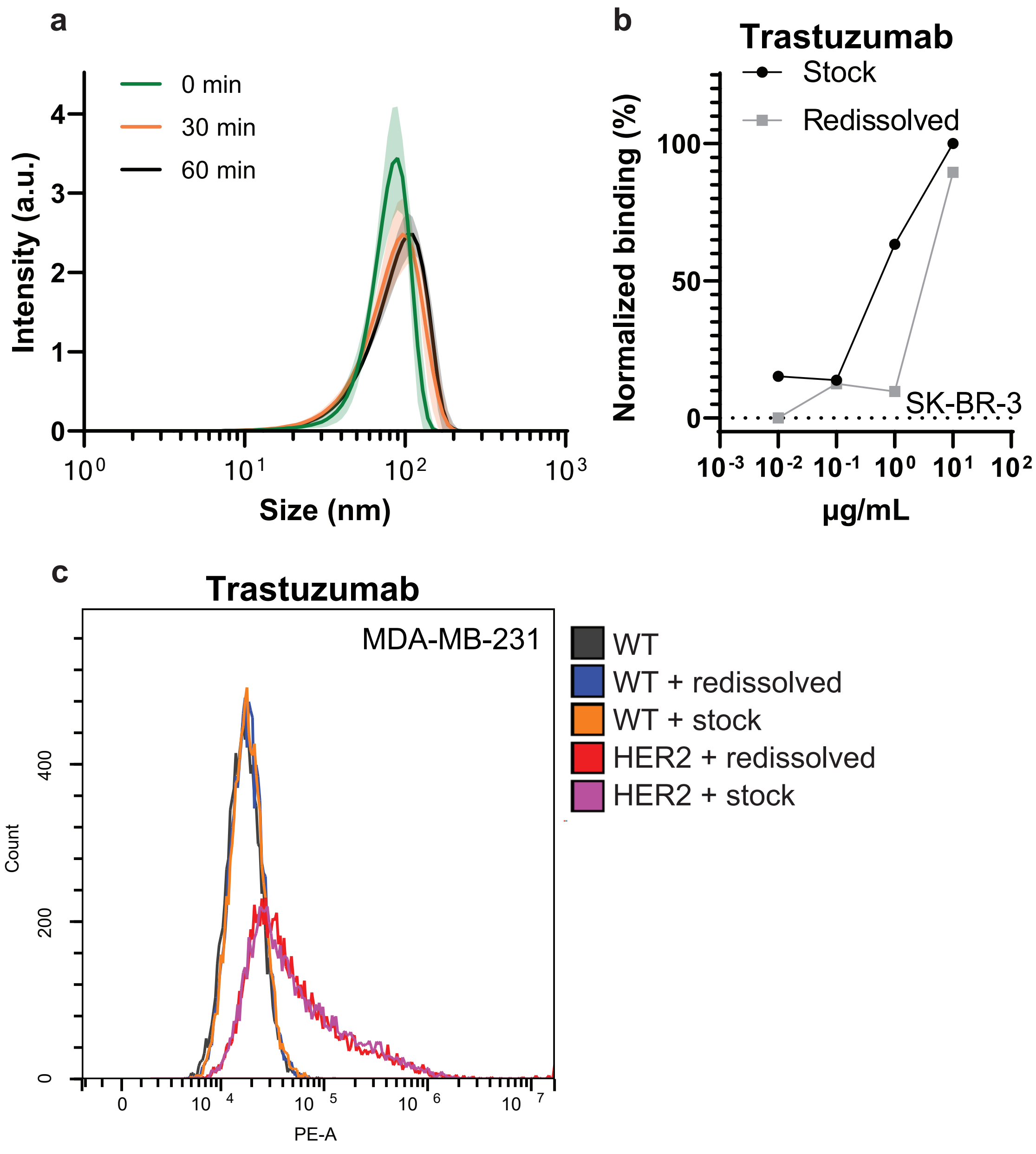

S1 Fig. Stability desolvated particles over time and cellular binding of redissolved trastuzumab. **(a)** Dynamic light scattering (DLS) intensity distribution of desolvated trastuzumab particles 0 min (n=4), 30 min (n=2) and 60 min (n=2) after production. **(b)** Binding study of redissolved and stock trastuzumab to SK-BR-3 cells, detected by recombinant FcR (n=1). **(c)** Histogram of redissolved and stock trastuzumab binding MDA-MB-231 and MDA-MB-231.HER2 cells (n=1). Data are presented as mean with all shaded areas denoting standard deviation.
